# Adamantinomatous craniopharyngioma: evolution in the management

**DOI:** 10.1007/s00381-023-06143-4

**Published:** 2023-09-20

**Authors:** Luca Massimi, Davide Palombi, Alessandra Musarra, Federico Bianchi, Paolo Frassanito, Gianpiero Tamburrini, Concezio Di Rocco

**Affiliations:** 1 Pediatric Neurosurgery, Fondazione Policlinico Universitario A. Gemelli IRCCS, Largo A. Gemelli, 8, 00168 Rome, Italy; 2 Department of Neuroscience, Università Cattolica del Sacro Cuore, Rome, Italy; 3 International Neuroscience Institute, Hannover, Germany

**Keywords:** Adamantinomatous craniopharyngioma, Management, Quality of life, Endoscopic endonasal transphenoidal surgery, Proton therapy, Intracystic therapy, Interferon alpha

## Abstract

**Background:**

In spite of the continuous progresses in pediatric neurosurgery, adamantinomatous craniopharyngioma (AC) remains a challenging tumor due to its proximity to optic pathways, pituitary gland, hypothalamus, and Willis’ circle, which can result in significant endocrine, cognitive, and neurological morbidity after treatment with subsequent impact on the patient’s quality of life (QoL). The relevance that QoL has today explains the changes in the management of AC observed over the time. The goal of the present article is to provide a historical background, to show the milestones in the changes of the AC treatment, and to analyze the current main options to manage such a challenging tumor.

**Material and methods:**

The pertinent literature has been reviewed. Moreover, a comparison between the past and recent personal series is reported.

**Results:**

Three main eras have been identified. The first (named Cushing era) was characterized by the need to realize a harmless surgery and to define the best way to approach AC; the second (microscope era) was characterized by a tremendous technical and technological development, with remarkable results in term of safe tumor resection and control but relatively poor QoL outcomes; and the third one (current period) is characterized by an increasing integration between surgery and adjuvant treatments, with relatively minor tumor control but significant improvement of QoL (comparable overall survival). The authors’ experience reflects these changes. Two groups of children were compared: 52 cases (mean follow-up: 17.5 years) belong to the historical series (group 1, 1985–2003, aggressive surgical management) and 41 (mean follow-up: 8.5 years) to the current one (Group 2, 2004–2021, integrated management). No significant differences between the two groups were detected about recurrence rate, surgical mortality, and overall survival. However, Group 2 showed significant lower rates of postoperative panhypopituitarism, obesity, and visual deterioration.

**Conclusions:**

Radical surgery allows for a good AC control with a low rate of recurrence but high risk of permanent morbidity. Despite the greater number of recurrences and surgeries, the more conservative policy, based on a combination of treatments, seems to provide the same tumor control with a better QoL. The advances in trans-nasal and trans-ventricular endoscopy, in proton therapy and in the management of the AC cyst are the main factors that allowed such an improvement.

## Introduction

Adamantinomatous craniopharyngioma (AC) is a rare brain tumor classified by the World Health Organization as a tumor with low-grade malignancy, arising from remnants of the craniopharyngeal duct epithelium [1]. It accounts for 1.53–2.92/100,000 cases/year in children under 15 years and for 5–10% of sellar tumors in the pediatric population, representing 1.2–4% of all childhood intracranial tumors [2]. In spite of the “benign” pathological appearance, AC is a challenging disease because of the clinical and surgical implications (involvement of the optic-hypothalamic region and the Willis’ circle), thus rising a great interest among neurosurgeons since the birth of neurosurgery. Surgery still is the main treatment tool, cystic aspiration, intracystic chemotherapy, and irradiation being alternative options in selected patients. The increasing understanding of the AC genetic basis hopefully will provide targets for medical therapy. Anyway, a long-term multidisciplinary management is required for the follow-up, aiming at managing the possible surgical complications, the hypothalamic morbidity, the visual deficits, and the cognitive and neuropsychological sequelae that can impair the patient’s quality of life.

In this paper, the evolution of the management of AC is analyzed with particular emphasis on the main changes occurred in the different eras and on the new therapeutic options.

## Background

### The reasons of the challenge

As known, Harvey Cushing was used to refer to craniopharyngiomas as “the most forbidding of the intracranial tumors” [3]. This statement is still shareable. Actually, due to its proximity to highly eloquent (optic pathways, pituitary gland, and stalk) or vital structures (hypothalamus, Willis’ circle), both the growth and the treatment of AC can result in severe neurological, visual, and/or endocrine sequelae that can impact on quality of life (QoL), especially in long-term survivors. Indeed, 35% of AC patients can show already at the diagnosis a combination of symptoms related to hypothalamic dysfunction (increased body weight in 12–19%, behavioral changes, altered circadian rhythms, sleep irregularities, and imbalanced regulation of the body temperature, thirst, heart rate, and blood pressure) [4]. Postoperatively, moreover, hypothalamic dysfunction (mainly, severe obesity) can occur in a high number of patients, thus representing the main comorbidity. Despite an adequate hormonal substitution therapy, weight gain can present as well and is frequently exacerbated by other factors that limit physical activity. Visual impairment (reduced visual acuity and visual field with bi-temporal hemianopsia) can be detected in up to 50% of cases at the diagnosis [4]. Neurological sequelae, on the other hand, are more frequently observed in the postoperative period, including hemiparesis, epilepsy, cranial nerve deficits, and manifestations of cerebrovascular disease. In spite of the mainly temporary occurrence of these sequelae, long-term neurological deficits are found in about 8% of patients, with up to 35% of cases in children harboring huge AC [5].

**Fig. 1:**
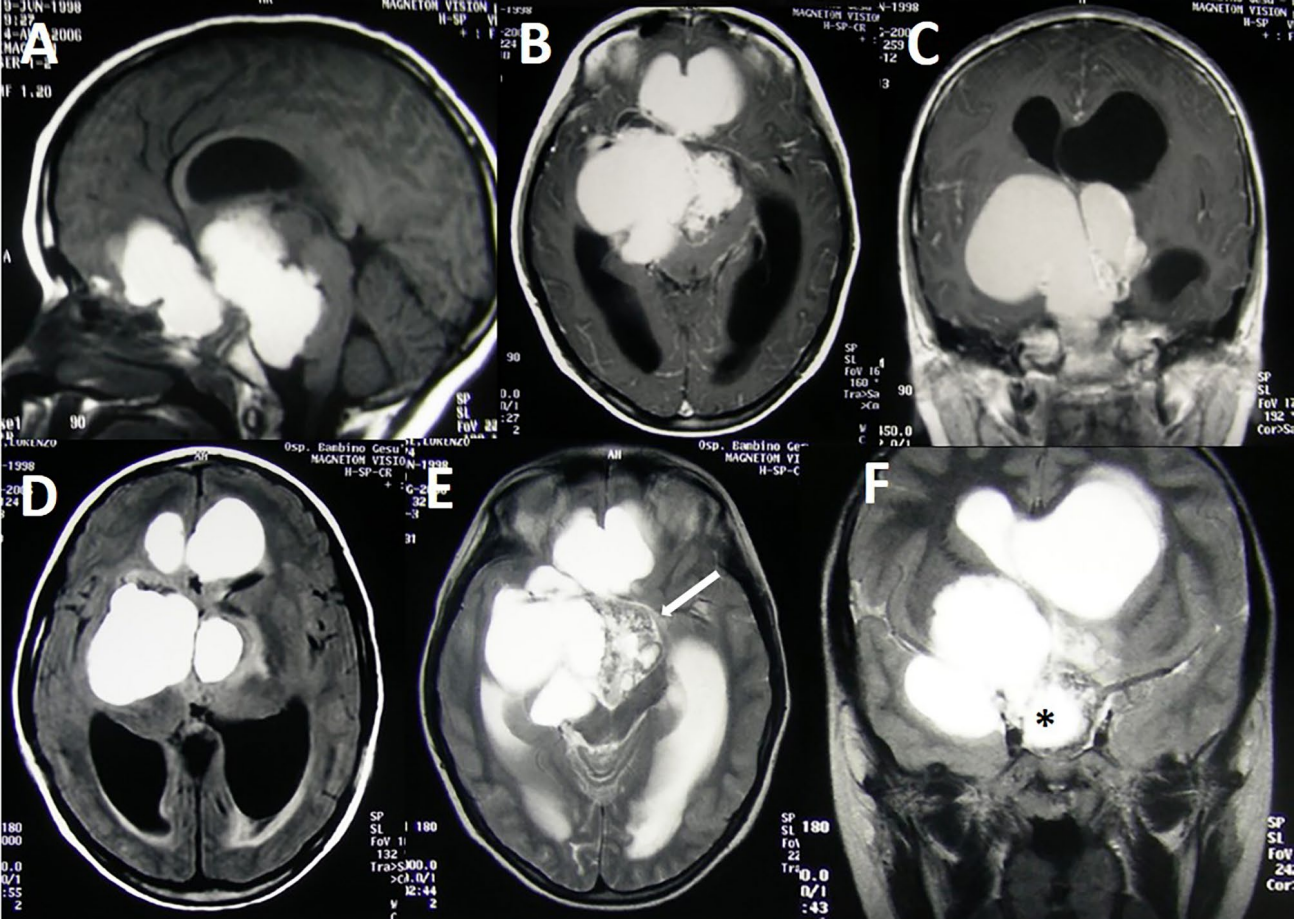
Giant AC in a 11-year-old boy. Note the large and enhancing cystic components in the T1 sequences after gadolinium administration (**A**, **B**, **C**), which extend into the anterior, middle, and posterior cranial fossa. Also a biventricular hydrocephalus is evident (**A**–**E**). The cyst fluid is hyperintense compared to CSF in FLAIR sequence (**D**). Note that the solid component is a minor part of the tumor (**E**, arrow) but involves the region of the Willis’ circle (**F**, asterisk)

The matter is complicated by the fact that surgery is the main therapeutic option, but, especially if compared with the papillary variant of craniopharyngioma, the complete surgical excision of AC is very hard to be obtained because of the possible low age of the patient, the often huge tumor size, the multi-cystic components, the encasement or the dense adhesions with the neighboring structures, and the presence of calcific portions often stick to the optic chiasm and the carotid artery (Fig. 1). In addition, AC is particularly prone to recurrence, even after an apparently gross total resection (GTR) [6].

### Evolution of treatment options

The first operation for craniopharyngioma we are aware of was performed more than one Century ago by Halstead in Chicago on July 1909 [7]. The author was unaware that the tumor was not an “ordinary” pituitary adenoma, and during surgery, he detected a cyst containing cholesterol crystals. After the operation, the patient’s vision improved. Since that time, the treatment management of AC showed epochal changes, mainly resulting from the technical and technological development and, more recently, the analysis of the results. According to Castro-Dufourny, 4 main eras in AC management can be recognized: (1) prior to 1970: in this era, loupes/headlight were the tools used for the surgical operation, brain RT was based on the sixties rudimental techniques, and the outcome was measured by the analysis of the morbidity and mortality rates; (2) 1970–1990: in this next generation, the introduction of the microscope and microsurgical techniques gave a significant input to the surgical management of AC. 2D conformal RT opened the modern era of radiation treatment and the GOS (Glasgow Outcome Score) started a new type of outcome assessment; (3) 1990–2010: the modern era was enriched by important technical advances, such as the development of endoscopic skull base surgery, or new kind of treatments, like the intracystic therapies, while RT evolved into the 3D conformal treatments. The outcome was assessed by general outcome scales (e.g., Karnofsky scale); (4) present time: in the current time, finally, the maintenance of a good QoL has become a crucial goal of the treatment and a way to assess the outcome; therefore, it is evaluated by specific scales and by neuropsychological tests. The treatment is tailored according to the patients’ characteristics taking into account not only the effectiveness of the therapy but also its side effects (the protons therapy summarizes the possibility to maximize the efficacy of a treatment without increasing the complications) [8].

The evolution of the diagnostic tools (X-rays, ventriculography, and angiography in the early phases, CT scan and standard MRI afterwards, and refined MRI with functional studies other than augmented reality simulation programs in current times) went with the aforementioned changes in the management and are strictly related to them.

In the following paragraphs, the main shifts in the AC management are summarized according the main technical and technological advances.

### The beginning and the Cushing’s experience

The first description of AC was probably provided by Erdheim in 1904, a pathologist from the General Hospital of Vienna, who performed an autopsy on a woman who died from cardiac insufficiency [9]. The author found a cyst on the anterior surface of the pituitary gland, whose wall was lined by squamous epithelium, different from the Rathke’s pouch. Therefore, the author concluded that the origin of this cyst was from vestiges of not involuted hypophyseal–pharyngeal duct and named it as “hypophyseal duct tumor.” In 1932, Cushing first used the term “craniopharyngioma”: ‘‘This admittedly somewhat cumbersome term has been employed, for want of something more brief to include the kaleidoscopic tumors, solid, and cystic, which take their origin from epithelial rests ascribable to an imperfect closure of the hypophysial or craniopharyngeal duct [10]. The Cushing’s series accounted for 124 tumors with the characteristics of craniopharyngioma. He performed 198 surgical operations in the patients of his series who underwent a neurosurgical treatment, with a 23% mortality rate calculated within the first 2 months from surgery. Also the other experiences in the same period were characterized by high mortality rates, ranging from 29 to 41% [11–14]. The Cushing’s career on craniopharyngioma surgery can be divided in 3 main periods which anticipate the need to progressively develop the management of such a challenging tumor (Table 1): early period, characterized by the use of the transsphenoidal approach and a limited drainage of the cystic component of the tumor; intermediate period, in which the subfrontal approach was adopted to attempt a maximal tumor removal; and late period, characterized by the use of air ventriculography for topographical diagnosis and limited tumor resection via transventricular approach. In spite of the highest rates of radical tumor resection and the good outcome related to the subfrontal approach period, Cushing was not completely satisfied about that. Actually, postoperative symptoms related to hypothalamic dysfunction occurred in 53% of cases. The removal of AC with third ventricle invasion was associated with the highest rates of hypothalamic injury. Among the complications, indeed, severe hypothalamic disturbances (malignant hyperthermia, impairment of consciousness, severe electrolyte imbalances) occurred in 18% of cases as consequence of traumatic or ischemic injury of the hypothalamus [15]. Cushing realized the importance of preserving the pituitary stalk and the hypothalamus. His legacy was received by the development of microsurgery or, more recently, by the extended transsphenoidal approaches [16, 17].

**Table 1:** Summary of the 3 periods of Cushing’s approach to AC

Period/year	Patients	Approach (cases)	Resection	Intraop complication	Outcome
Early: 1901–1917	39	Transphenoydal (14) Decompressive temporal craniectomy (11) Subforntal (10) Subtemporal (3) None (1)	GTR (1) PR (4) Biopsy (2) Cyst drainage (7)	Failed Surgery (3) Meningitis (3) HypoInjury (3) Heart arrest (1) CSF leak (2) Bleeding (2) None (23)	Good (14) Fair (8) Poor (6) Death (8)
Intermediate: 1919–1925	42	Subfrontal (33) Transphenoidal (6) Transventricular (1) Transcallosal (1) Cyst puncture (1)	GTR (7) SR (14) PR (11) Cyst drainage (8) Biopsy (1)	Meningitis (1) HypoInjury (10) Postop DI (2) Heart arrest (2) CSF leak (2) Bleeding (4) None (20)	Good (23) Fair (6) Poor (5) Death (6)
Late: 1926–1932	43	SubFfrontal (32) Transventricular (5) Decompressive temporal craniecotmy (1) Cyst puncture (1) None (4)	GTR (2) SR (9) PR (20) Cyst drainage (6) Biopsy (1)	Failed Surgery (1) Meningitis (1) HypoInjury (8) Insipid diabetes (2) Heart arrest (2) CSF leak (2) Bleeding (4) None (13)	Good (26) Fair (4) Poor (4) Death (6)

In summary, this era showed that AC is a challenging tumor whose main treatment is the surgical excision. In this period, the main efforts were devoted in favoring anatomical studies and refining the surgical techniques to find the best surgical way to approach such a complex tumor. The final goal was to reduce the excessively high rates of surgical mortality and morbidity.

### The changing management of AC

The “modern” eras are characterized by the advent of new concepts on neurosurgical techniques and new diagnostic and surgical technological tools that significantly contributed to improve the management of AC. Such a pathway has been covered together with the parallel development of medical and radiation therapies.

It is worth noting that the beginning of the microscope era coincided with the birth of the International Society for Pediatric Neurosurgery (ISPN). As a result, large part of evolution and changes in the AC management were due to ISPN members.

### The microscope era: “The more the better”

With the introduction of the operating microscope, a new management strategy started to be adopted in many centers: the GTR of the tumor. Thanks to the operating microscope, the wall of AC was more safely separable from the surrounding vessels and the chiasm, thus reducing the risk of ischemic complications [18, 19]. Due to the contemporary improvements in the anesthesiology and intensive care management, GTR in childhood became more and more possible without significant surgical mortality and with a limited neurological morbidity. At the same time, the development of reliable substitutive drugs provided a further impulse in enlarging the surgical limits thanks to the possibility to support adequately the patients with postoperative hormone imbalance. The histologically benign features of AC, finally, theoretically made it an ideal target for curative radical surgical resection, namely with the goal of preserving the visual function and field [17, 19–22]. In 1969, Matson et al. reported on series of 57 patients where GTR was obtained in 77% of cases: the authors emphasized that the ideal treatment of AC in children should consist of making every efforts to achieve GTR at the first operation, followed by conscientious regular endocrine and neurological evaluation [15]. Hoffman et al. “took to extremes” this surgical strategy by achieving a 90% GTR in their 50 patients and a SR in the remaining 10% [20]. The authors had one surgical death (2%). Most of postoperative morbidity occurred after the second or third operation (among them, weight gain, hyperosmolality, panhyipopituitarism, severe and prolonged convulsion, water intoxication occurred). These reoperations were necessary because, in spite of the high rate of GTR, 34% of AC recurred after a 32.6-month mean period. Among 46 patients with complete follow-up, 60% had a normal or nearly normal life.

The magnification due to the operating microscope, the resectability of AC and the fear for a possible recurrence led to consider GTR so mandatory that even the voluntary excision of the pituitary stalk was justified to avoid AC remnants [23]. It is worth noting that such an “aggressive” policy did not result only by the need to obtain GTR but also from the evidence of a very high rate of children with endocrine dysfunction already at the diagnosis [24]. The progresses in the neuroimaging techniques other than in microsurgery increased the recognition of the appropriate surgical approaches, thus enhancing the surgeon’s ability to attain GTR in AC and explaining the peak of enthusiasm that craniopharyngioma surgery reached at the beginning of 1990s. Such an enthusiasm was justified by the significant drop of the tumor-related mortality (up to 50% at 10 years before the aggressive strategy) and the tumor recurrence (up to 50% in some series) [17, 20, 21, 25–37].

Unexpectedly, even the endoscopic techniques started to evolve in this rich historical period. AC was actually considered ideal to be reached through the transsphenoidal approach since the beginning. However, the first surgical approaches to the pituitary region were extensive and disfiguring; other than burdened by a significant morbidity [38]. The introduction of transcranial microsurgery gave a significant input also to the evolution of transsphenoidal surgery, favoring more precise, narrow, and effective approaches. Starting from the 1960s, several technical and technological tools were introduced to enhance the transsphenoidal route: Dott and Guiot started using lighted nasal speculums and intraoperative image intensification and fluoroscopy and Hardy introduced the binocular microscope [39]. Finally, Apuzzo and coworkers, in 1977, described the use of a side-viewing endoscope to provide access to angles hidden from the microsurgical approach [40]. These advances forewarn the endoscopic renaissance (both ventricular and endonasal) that will characterize the next era.

In the meanwhile, similar progresses were being obtained in radiotherapy techniques. From 1970s, the linear accelerator started replacing the 60Co radiation therapy (RT), and due to the diffusion of CT scan, the calculation of the radiation field became more precise. Both these advances allowed the clinicians to evaluate the tumor response to RT more precisely. Therefore, it was evident that external-beam photon RT was effective in attenuating tumor growth and preventing recurrences but, at the same time, also the damages resulting from the wide irradiated brain area were evident, especially in children. Already in 1961, Kramer et al. published a report on the role of limited surgery followed by RT in 6 children with AC treated at the Royal Marsden Hospital between 1952 and 1954 (combined surgery and external beam radiation therapy delivering 5500 Roentgen over 6 weeks) [41]. RT was delivered along the sella turcica and at level of the calcifications beyond the sella, based on X-rays. All patients were alive and free from recurrence after 6 years. During the following years, the series undergoing conservative surgery and adjuvant RT at the same institution progressively enlarged [36, 42, 43]. More recent series (between 1980 and 2000) confirmed the good control of the disease, with a progression free survival ranging from 79 to 84% [29, 32, 44–46]. It is not surprising, therefore, to find data supporting the reliability of partial resection of AC plus adjuvant RT compared with GTR in the era of the maximal surgical resection strategy. Nevertheless, since RT was not so developed to result curative at that time, to make any efforts to remove AC at the first operation was the main suggestion given by experienced neurosurgeons [47].

In summary, this era was characterized by a stimulating research on the way to maximize the surgical excision and the overall survival of patients with AC (Table 2) [17, 20, 27, 28, 34, 36, 48–88]. Actually, the tumor recurrence/regrowth appeared as the worst complication in the management of this tumor while, at the same time, the concept of caring the QoL was not so developed yet (e.g., note the high rate of not available data on obesity in Table 2). As a result, the technical and technological progresses (not only in neurosurgery but also in the general care of patients), which made surgery safer and more effective, allowed to push more and more ahead the limits of surgery, in spite of the risk of postoperative complications. However, always during this period, the improvement of the adjuvant therapies and the introduction of the evaluation of the functional outcomes laid the foundations for changes characterizing the next era.

**Table 2:** Summary of the main experiences during the microscope era and the transition to the current period, with postoperative results and outcomes (modified from 52)

Authors (reference)	Year	N. of pts	GTR (%)	Neurological morbidity (%)	Insipid diabetes (%)	Obesity (%)	Surgical mortality (%)	Overall Survival (%)
Colangelo et al. [51]	1990	32	22	12	NA	NA	12	75
Fischer et al. [28]	1990	37	19	NA	34	NA	0	92
Yaşargil et al. [17]	1990	144	90	NA	90	NA	2.9	80
Samii and Bini [77]	1990	34	100	0	>80	NA	0	NA
Symon et al. [83]	1991	50	60	11	>50	8.7	4	76
Hoffman et al. [20]	1992	50	90	6	93	52	2	98
Tomita and McLone [34]	1993	27	78	NA	81	NA	0	93
Pierre-Kahn et al. [74]	1994	30	83	NA	NA	NA	0	NA
Maira et al. [68]	1995	22	71	4.5	NA	NA	0	86.4
Mark et al. [69]	1995	49	39	10	NA	NA	10	NA
De Vile et al. [27]	1996	75	40	13	80	15	0	88
Shibuya et al. [79]	1996	22	NA	7	97	NA	0	90
Bülow et al. [49]	1998	26	73	NA	NA	NA	8	88
Fisher et al. [57]	1998	30	27	NA	83	NA	0	93
Khafaga et al. [64]	1998	44	39	NA	NA	NA	16	73
Fahlbusch et al. [56]	1999	94	46	0	NA	6.7	0	91.5
Kim et al. [65]	2001	36	100	42	94	6	0	89
Merchant et al. [70]	2002	30	27	10	50	NA	0	97
Van Effentere and Boch [86]	2002	122	79	8	57	36	2.5	89
Chen et al. [50]	2003	36	19	14	67	NA	5.6	NA
Kalapurakal et al. [61]	2003	25	76	NA	100	32	0	100
Gonc et al. [58]	2004	66	33	10.6	52	NA	2	80
Stripp et al. [82]	2004	76	62	NA	80	49	1	89
Albright et al. [48]	2005	27	67	11	NA	NA	0	NA
Erşahin et al. [55]	2005	87	43	15	33	NA	7	89
Karavitaki et al. [62]	2005	42	17	12.9	65	39	0	80
Lena et al. [67]	2005	47	66	NA	86	48	2.4	94
Minamida et al. [71]	2005	37	70	2.7	NA	NA	0	94
Mottolese et al. [72]	2005	36	74	NA	100	NA	6	89
Sainte-Rose et al. [76]	2005	66	50	NA	NA	70	NA	NA
Shirane et al. [80]	2005	42	71	6.7	NA	NA	0	93
Sosa et al. [81]	2005	35	83	20	91	NA	0	97
Thompson et al. [84]	2005	48	33	15	84	20	0	96
Tomita and Bowman [85]	2005	54	61	9	87	28	0	90
Zuccaro et al. [36]	2005	153	69	10	50	35	3	87.5
Xu et al. [87]	2005	51	92	8	NA	NA	0	94
Dhellemmes and Vinchon [52]	2006	37	65	8	NA	48	0	89
Di Rocco et al. [53]	2006	54	78	18	56	18	3.7	93
Gupta et al. [59]	2006	72	26	NA	NA	NA	NA	NA
Hafez et al. [60]	2006	62	50	6	85	NA	6	NA
Ohmori et al. [73]	2007	27	93	NA	NA	NA	3.7	93
Puget et al.* [75]	2007	66	50	6	94	70	1.5	94
Puget et al.** [75]	2007	22	23	14	100	27	0	100
Lee et al. [66]	2008	66	NA	6	66.7	18	0	97
Shi et al. [78]	2008	309	89.3	6	53	NA	3.9	94
Zhang et al. [88]	2008	202	40	5.4	81	22	1	<70
Elliot et al. [54]	2010	57	100	12	78	15	3.5	93
Kawamata et al. [63]	2010	55	22	NA	45	NA	0	89

*NA* not available

^*^Retrospective series; ^**^Prospective series

### How to bright the dark side of moon: the surgical pendulum era

The search for GTR showed the “dark side of the moon” of AC surgery with the recognition of too high rates of complications and relatively high rates of recurrence even after an apparently GTR [28, 35, 108]. Subsequently, this era, which started at the beginning of the current century and still lasts, is characterized by the experimentation of new management solutions and different combination of treatments, the main goals of these treatment being to preserve the patient’s QoL. Indeed, the developing age requires efforts to safeguard the physical, sexual, and psychomotor development of children affected by AC. Therefore, strategies favoring limited surgical excision of AC followed by RT or options encompassing temporary measures to postpone the surgical operation (e.g., use of intracystic drugs) have been more and more used during this time period. Right now, just at the end of this well-balanced era, there is certain trend to go back to a prevalently surgical management of AC (probably because of the improvement in endoscopic endonasal surgery). The most important and probably most recent acquisition of this time period is to consider and manage AC as a chronic disease, providing solutions not only to minimize the treatment complications but also for long-term clinical and neuropsychological interventions [109]. Actually, the survival analysis in large series, demonstrated that, unlikely most brain tumors, the extent of resection does not critically affect the overall survival in AC [110]. The Kraniopharygeom 2007 project can be mentioned as a summary of this period. Such a German project, indeed, had/has the goal to study AC from 2007 in all its main features to provide information and increase the knowledge on the outcome [111] and about very specific aspects, like cerebral infarction [112] or cardiac remodeling in AC [113] or pregnancy after childhood onset AC [114].

Among the several centers that emphasized the need to bright the dark side of the moon, the Catholic University’s group in Rome and the Necker’s group in Paris published focused articles on this subject. In his personal communications, done during the previous era and often criticized at that time, Di Rocco first started questioning the need to obtain GTR at any cost in AC. Actually, several subsequent studies, carried out with his group, clearly demonstrated that surgery for AC was feasible and GTR could be obtained without a significant risk of mortality and permanent neurolgoical morbidity, but the weight of the hypothalamic dysfunction was not more acceptable according to the modern standard of QoL [5, 53, 91]. These studies showed the changes in the management policy of the Rome group over the time, shifting from an aggressive surgical management to a less aggressive approach tailored on the patient’s characteristics: GTR in AC with poor hypothalamic involvement and old children/adolescents, subtotal or even partial resection followed by RT in case of hypothalamic involvement, and use of intracystic interferon-alpha to postpone surgery and RT as much as possible in young children. In the same period, Sainte-Rose and the Necker’s group, by reviewing their 20-year long experience, found that QoL of AC patients correlates with the degree of hypothalamic damage evident on the postoperative MRI [76, 115]. The authors proposed a multimodal treatment based on the possibility to predict the hypothalamic damage according to the degree of hypothalamic involvement on preoperative MRI. According to the Puget’s classification, such an involvement was classified in 3 types: type 0: no involvement of hypothalamus (GTR is suitable); type 1: compression but not invasion of the hypothalamus (GTR can be still attempted); and type 2: hypthalamus invasion (limited surgical excision followed by RT) [75]. Although questioned because of the poor correlation between MRI and intraoperative possibility to detect a hypothalamic invasion, this classification suggested the need to let the surgical pendulum swing from an aggressive to a conservative management. The most important argument against the conservative management came from the increase of tumor recurrences (often 100%) so that, since the beginning of the new era, the need of a personalized approach to AC was emphasized [61]. As showed by Boop in the Saint-Jude’s experience, a personalized approach, including conservative surgery (aimed at decompressing the optic pathways by leaving intact the arachnoid plane for a possible second look surgery) and conformal RT was the best solution to preserve both the tumor control and the QoL [116].

A great support to such a swinging of the pendulum was related to the advances in neuroimaging and conformal RT that led to a continuous refinement of the techniques to localize the tumor and limit the dose of radiation delivered, making more suitable the goal to reduce neurocognitive and endocrinological damages and to improve the patients’ QoL. The benefit in reducing all the RT complications (visual deterioration, optic neuropathies, vasculopathies, and Moyamoya disease, RT-related secondary tumors) was evident [117]. Moreover, the possibility to achieve the same long-term control of the disease than GTR resulted crucial to move several centers to prefer the partial/subtotal resection plus RT option [110, 118]. Yang and colleagues published a systematic review comparing the efficacy of different treatment options in more than 400 patients with craniopharyngiomas [110]. GTR was achieved in 58% of cases, subtotal resection (ST) in 23%, and ST and RT in 19%. The 5- and 10-year overall survival rates of GTR and STR+RT did not differ (98% versus 95%) as well as the progression free survival (67% versus 69%). As an evolution, more and more focused RT variants were explored to preserve cognition and improve patients’ QoL. Gamma knife radiosurgery (GKR) was realized to maximize the RT doses deliverable to the tumor by limiting those involving the surrounding tissue (namely, the optic pathways). GKR consists of the stereotactic administration of a single dose of 12–30 Gy or two to five fractionated doses, in case of near proximity with the optic pathways. Losa et al. proposed hypofractionated GKR to deliver high doses to the tumor and to reduce the risk of damaging the optic ways [119]. Ogino et al. found that the tumor control can be maximized, by reducing the risk of complication at the same time, when > 85% of AC receives at least 12 Gy [120]. More in details, the study included 53 patients undergoing a single-session stereotactic radiosurgery for recurrent or residual AC, when ≥ 85% of the tumor received ≥ 12 Gy, the tumor control rates at 3-, 5-, and 10-year were 100%, 93.3%, and 93.3%, respectively.

A further, relevant evolution of RT was represented by intensity-modulated radiation therapy (IMRT), once again designed to spare surrounding structures from high doses of radiation and toxicity (merchant 2022). In the management of AC, IMRT showed no significant difference in PFS (65.8% at 5 years) and OS (96% at 5 years) rates compared with 2D and non-IMRT 3D RT techniques, the solid component demonstrating a better response than the cystic part [121–123].

The most recent and promising evolution of RT is the proton-beam therapy (PBT), which allows the patient to receive a greater dose distribution with reduced risk of injury of the surrounding, normal tissues taking advantage of the different radiation physics of the particle beams and the different deposition of the radiation dose in the tissues. The final result is the reduction of brain irradiation, especially on the temporal lobes [104]. PBT is administered by means of charged particles with mass which concentrate the dose on the target with only a small amount of energy being delivered along the particle path. Merchant et al. provided a large analysis of long-term outcome in a series of 94 children with AC undergoing PBT [100] who were compared with their historical series of 101 pediatric patients treated by photon RT [99]. Taking into account the limitations of the heterogeneous protocols characterizing the historical series, the authors found similar results between their two series as far as survival outcomes and severe complications (endocrine deficits, vasculopathy, neurological, or visual deficits) were concerned. However, a significant decrease in neurocognitive dysfunction was detected compared to conventional radiotherapy. The still limited availability and the high costs are the main limits of PBT [104].

The concept of maximal safe resection plus RT and the concomitant advent of endoscopic transshpenoidal surgery (which seemed to be more promising than transcranial surgery about the safety of resection) raised a great debate on the use of the transcranial microsurgical approach (TMA) versus the endoscopic endonasal transsphenoidal approach (EETA) [89, 124]. Such a debate was really common during this era. Initially, it was in favor of TMA because EETA was limited to sellar or suprasellar, infradiaphragmatic AC, preferably in patients with a well-pneumatized sphenoid sinus and enlargement of the pituitary fossa [124]. Afterwards, EETA, which was more and more diffused and technically improved, gained a relevant consent because of some obvious advantages (direct access to the tumor, no brain manipulation) [125, 126]. At present, finally, it has been realized that these two approaches are both effective but should be performed in different population of children, alone (EETA in patients with mainly sellar/suprasellar midline AC while TMA in patients with large and laterally extending AC) or even in combination (huge AC) [6, 89].

As mentioned, EETA could have contributed to a certain renaissance of the surgical excision of AC in the current times. Actually, EETA was initially dedicated to the skull base lesions in adults and became very popular thanks to the absence of brain retraction, the faster recovery and the shorter hospital stay, the absence of a visible surgical scar, and the direct endoscopic visualization (close to the tumor and possible also on hidden angles) compared with TMA [127–132]. The major limitations of EETA, which are represented by tumors with lateral and superior extensions, limited possibility to repair directly microvascular damages and CSF leakage have been overcome by the introduction of extended approaches, the learning curve, and the specific reconstruction techniques, respectively [16, 133, 134]. A recent multicentric, Italian study analyzed the impact of EETA in adult and pediatric infradiaphragmatic craniopharyngiomas (84 patients treated between 2000 and 2021 in 6 different centers) [135]. A good rate of GTR (72%) was detected in case of tumor involving the region below the chiasmatic cistern together with a satisfactory visual outcome (improvement in 75%) confirming the safety and efficacy of this approach. GTR in tumors extending above the chiasmatic cistern was lower (54%) but visual function improved in the majority of cases even in this group (72%). The most common complication remained CSF leakage (14%). Just to remain on the recent literature, Cao Lei et al. reported about a very large series of 182 patients (both children and adults), treated at a single Institution, and compared EETA with TMA [136]. The EETA group showed higher GTR rate than TMA (97% vs 61% for intrasellar tumors and 93% vs 77% for suprasellar ones), lower incidence of new onset hypopituitarism (69% vs 32%), and better visual outcome. About the latter: in case of intrasellar AC, an improvement was detected in 60.3% of cases (36 patients with EETA and 5 with TMA) and a deterioration in 10.3% (3 patients with EETA and 4 with TMA); for suprasellar AC, an improvement occurred in 60.5% of cases (31 patients with EETA and 15 with TMA) while a deterioration in 14.5% (4 with EETA and 7 with TMA). No significant difference in the incidence of diabetes insipidus was found between the 2 groups (23–32%). The authors concluded that EETA should be considered as the first-line surgical modality for craniopharyngioma.

The theoretical limits of EETA in the pediatric population, manly related to the different anatomic conditions (e.g., small nostrils, poorly developed paranasal sinuses, growing bony structures) or to the lack of a dedicated surgical instruments have been overcome too thanks to the refinement of the technique and the technology so that EETA is more and more used to approach anterior skull base tumors in children or even those located in the third ventricle [97, 101, 137]. According to a personal experience comparing EETA with sub-labial microsurgery (31 children, 35% with AC), a significant difference between the two approaches was found about admission to ICU (35% vs 100%), blood transfusion (23% vs 71%), duration of hospital stay (4 vs 5.7 days), and pain perception scores (2.05±0.74 versus 2.92±0.91 in the early postoperative course, 0.82±0.95 versus 1.64±0.84 in the late one) [138]. No differences in term of early complications and functional or oncological outcome were found. Therefore, EETA resulted more advantageous than microsurgery as far as the trans-sphenoidal route is concerned. More recently, Mazzatenta et al. analyzed a series of 25 consecutive cases of pediatric AC treated by EETA, showing a very high rate of GTR (92%) and normalization/improvement of visual function (43%) [139]. On the other hand, complication as postoperative endocrine dysfunction and CSF leak remained as high as 92% and 24%, respectively. The recurrence rate was 19%. These figures demonstrated that EETA is a reliable approach in children too but also that, in spite of the effectiveness the technique, AC remains a challenging tumor as far as complication and risk of recurrence are concerned. Another implication of EETA concerns the reduced efficacy in case of tumor recurrence. Indeed, the possibility to achieve GTR is higher at the time of the first operation than at the time of the tumor recurrence [107] although such a difference may be not statistically significant [130].

A further contribute to the recent surgical “revival” comes from the evidence of long-term effects of RT on the neurocognitive development. Actually, recent studies did not show differences between GTR and partial resection (PR) plus RT as far as intellectual outcome and QoL scale scores were concerned, GRT being even superior in term of improvement in adaptive behavior and conceptual skills [140]. It is worth noting, however, that proton RT maintains the same neuro-psychological outcomes than GTR but with a minor rate of hypothalamic dysfunction [99, 141].

### Overview of the current options for the management of the cyst

The AC-related cyst deserves a special mention for several reasons. First, AC is mainly characterized for the presence of a predominant cystic component (about 90% of cases) [142]. Second, the surgical excision of the whole cyst is often impossible because of the adherence of the cystic wall to vessels, optic ways, and third ventricle. Therefore, the risk of cyst recurrence remains a challenge. Third, RT is effective on the solid part of AC while it shows an unpredictable but usually minor effect on the cystic component. Fourth, the often rapid growth of the cystic part may cause problems during RT due to the need to modify accordingly the irradiation field [96]. Fifth, fortunately, the cystic component may benefit from dedicated treatments. Most of these treatments were designed during the “surgery” era for unresectable or recurrent AC but are more extensively adopted during the current era to reduce the impact of surgery and RT [143].

### Cyst aspiration

The intracystic positioning of an Ommaya reservoir is one of the most common techniques, used for a cyst aspiration and/or to deliver drugs into the cyst itself. The placement of a catheter inside the cyst can be performed under direct vision, or under stereotactic or ultrasounds or ventriculoscopic or fluoroscopic guidance, as well as with stereotactic endoscopic technique [144, 145]. Currently, the navigated endoscopic transventricular approach is the most commonly used technique [146]. This approach, indeed, is mini-invasive (neuroendoscopy) and reliable (neuronavigation); moreover, it allows the surgeon to visualize directly the position of the catheter across the cyst and to perform additional surgical maneuvers (tumor biopsy, cyst aspiration, third ventriculostomy, septostomy), if required.

The fluid aspiration is the most “basic” approach to the cyst, being realized simply through an intracystic catheter connected to a subcutaneous reservoir (Rickham or Ommaya). It is used to obtain a quick tumor decompression and to re-aspirate fluid in case of cyst recurrence. As expected, this technique is a transient measure to be adopted to gain time for a more radical treatment in quickly growing AC or in case of poorly clinical condition. However, the cyst aspiration alone has been proved to be effective even in the long-term period. Actually, Moussa et al. reported that up to 70% of their patients did not show a cyst recurrence and did not need further treatments in a 7-year follow-up [147]. Only 19% of patients required a repeated procedure every 6 months and 8% of patients received further treatments.

In case of partially solid or giant AC, the cyst aspiration may be used as part of multimodal treatment involving TMA or EETA. The cyst drainage, indeed, can facilitate the surgical tumor removal by reducing the tumor volume and, therefore, can reduce the risk of complication [148–150]. Similarly, the fluid subtraction favors RT by removing a not radiosensitive component of the tumor and by reducing the irradiation field. Finally, such multimodal management may involve neuroendoscopy both for fluid aspiration and for tumor removal in case of AC mainly involving the third ventricle [151].

### Beta-emitting radionuclides

Thanks to their penetration width of 3–4 mm, the β-radiations are effective in damaging the cyst wall so that different β-emitting sources (Yttrium90, Rhenium186, Aurum198, or Phosphorous32) have been proposed to manage cystic AC. Phosphorus32 (P-32) is the most used one because if offers longer half-life, lower required dose and lower half-value tissue penetrance than other radionuclides [98]. The treatment with P-32 alone can induce a cyst reduction in up to 70–80% of cases in the short-term period, but it is burdened by a high rate of recurrence in the long-term one [152–154]. On the other hand, if used in combination with cyst aspiration, P-32 is able to stop the AC growth in to 75% of cases and to maintain the result for some years (mean follow-up reported: 48.6 months) [155]. In a recent series of 32 patients treated by P-32 for recurrent craniopharyngioma, Hu et al. identified 4 types of tumors according to the thickness of the cyst wall and the expression of vascular endothelial growth factor (VEGF) and VEGF receptor-2 (VEGFR-2) [156]. Thin cyst (type I and II) and expression of VEGFR-2 were predictive of a good response to P-32, whilst types III and IV (and no VEGFR-2 expression) were not.

The current use of radionuclides is limited by several factors, as their possible side effects, the complex protocols for their transport, handle and disposal, and their poor availability [157]. The most feared risk remains the spillover of the radionuclide into the cerebrospinal fluid, although its incidence and impact are hard to be assessed. According to one of the largest series (53 patients) published so far by Kickingereder et al., the risk of permanent complications of this treatment does not overcome that of other therapies, the permanent neurological and hormone deficits being observed in 4% and 2% of cases, respectively [94].

### Bleomycin

First discovered by Umezawa in 1966 and launched in Japan by Kayaku in 1969, such a “natural” antibiotic (bleomycin is a glicopeptide secreted by Streptomyces Verticillus) was proved to be effective against several epithelial tumors [158]. Therefore, the squamous epithelium of AC cyst wall was considered an ideal target for bleomycin since the beginning: Kubo obtained good result against cultured craniopharyngioma cells already in 1971 [106] and Takahashi reported on the first clinical series (7 patients) in 1985 where bleomycin was observed to be effective on the cystic component of AC but not on the solid portion [159]. Since there, it was more and more used with different protocols varying according to the different centers.

Several studies demonstrate the effectiveness of bleomycin in the transient control of the AC-related cyst. Hukin et al. analyzing their large experience on pediatric cases from 1982 to 2003 found that intracystic bleomycin definitely allows to postpone surgery as much as possible and should be preferred to other more invasive treatment in selected patients [160]. Mottolese and coworkers compared a group of 36 patients treated by direct surgical approach alone with a group of 24 patients managed by intracystic bleomycin administration (with or without surgery) and observed a significantly lower rate of morbidity and mortality in the bleomycin group [72]. In spite of these good results and the large cumulative experience in the literature, there is not enough evidence yet to recommend bleomycin to manage AC because of the heterogeneity of the series, the lack of randomized trials and the risk of adverse events [102]. Although carried out in a small sample of patient (7 cases), the randomized controlled trial by Jiang et al. on the comparison of bleomycin and P-32 alone and in combination demonstrated the superiority of the combination treatment in shrinking the cyst [161]. However, the authors acknowledged an important limitation of this approach in term of endocrine imbalance and other severe adverse events (bilateral thalamic infarction in 22% of cases). The main side effects of bleomycin are fever (up to 70% of cases), headache, and nausea, followed by delayed toxic effects due to damage to neural and vascular structures (transient neurological deficits, ischemic vasculopathy, permanent optic pathway, and/or hypothalamic injury [162–164].

### Interferon alpha

Interferon-alpha (IA) is a cytokine produced by macrophages and lymphocytes as response to viral infection which can have also an antitumoral activity because it promotes the differentiation of T cells into T-helper lymphocytes, it stimulates the proliferation on natural killer cells and macrophages, it provides the production of IL-1 and IA itself, and finally, it increases the expression of the MHC complex class 1 and surface antigens [165, 166]. As for bleomycin, the experience with IA started with skin epithelial tumors (squamous and basal cell carcinomas) that share the same origin with AC [167]. Its use on AC was favored not only by the epithelial origin of AC but also by the evidence of the predominant role of inflammation in the growth of AC-related cyst [168–171]. After the first, positive experience with the systemic (subcutaneous) administration of IA in reducing the AC growth, Cavalheiro et al. realized a trial on the intracystic use of IA, reporting on a complete response in about two thirds of children in a 1.7-year mean follow-up [145]. Other than the good initial results, the most important factor which contributed to the diffusion of IA was the occurrence of only mild adverse events (self-limiting headaches, fever, fatigue).

**Fig. 2:**
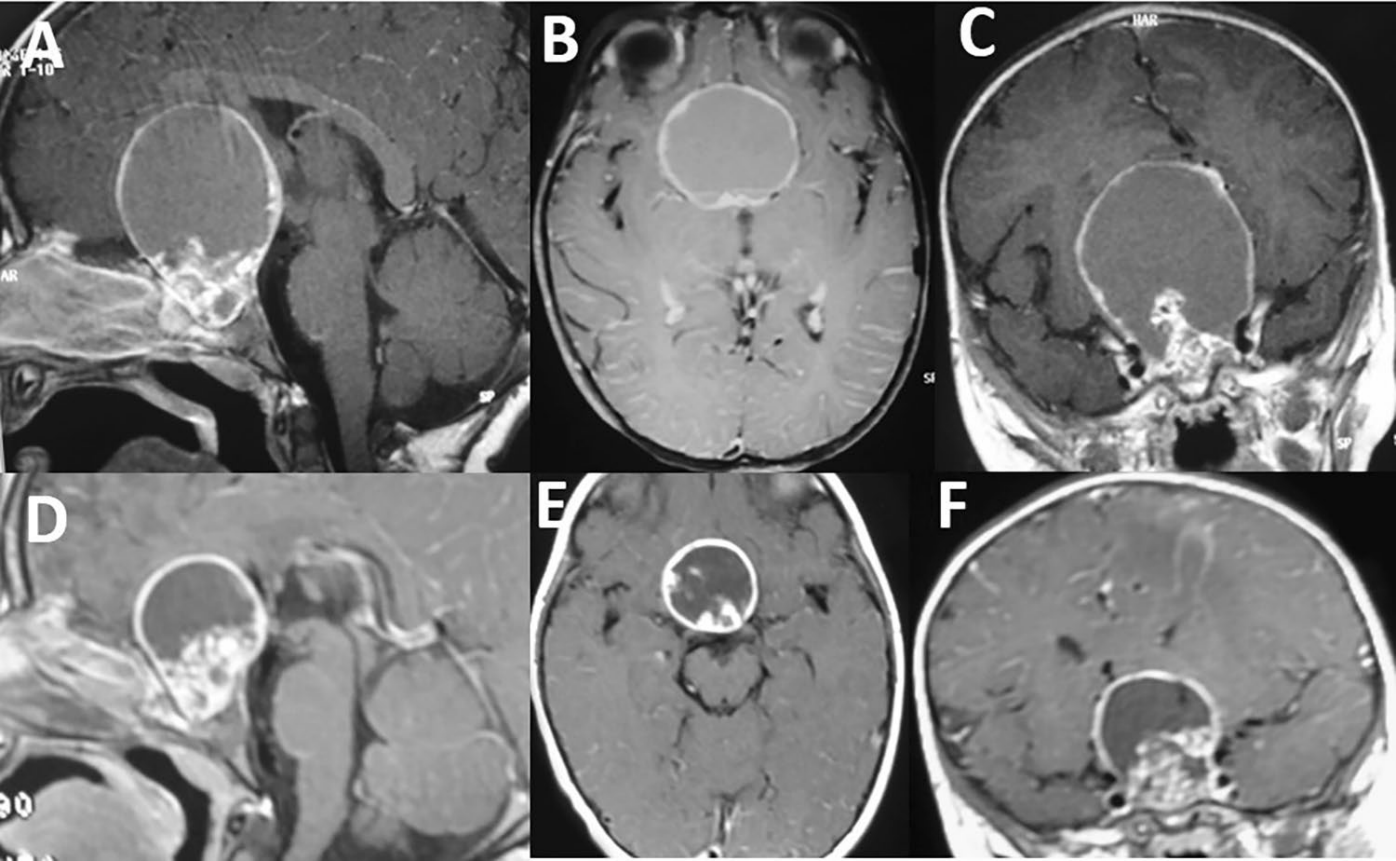
Pre-treatment T1 sagittal (**A**), axial (**B**), and coronal MRI (**C**) of AC in a 5-year-old girl. Note the relatively small, solid, sella portion, and the large suprasellar/third ventricle cystic component. The same sequences performed 2 years later (**D**, **E**, **F**), at the end of 3 cycles of IA, show a quite good control of the disease, with shrinkage of the cyst. The cyst wall remains thick and present a “rigid” appearance

**Fig. 3:**
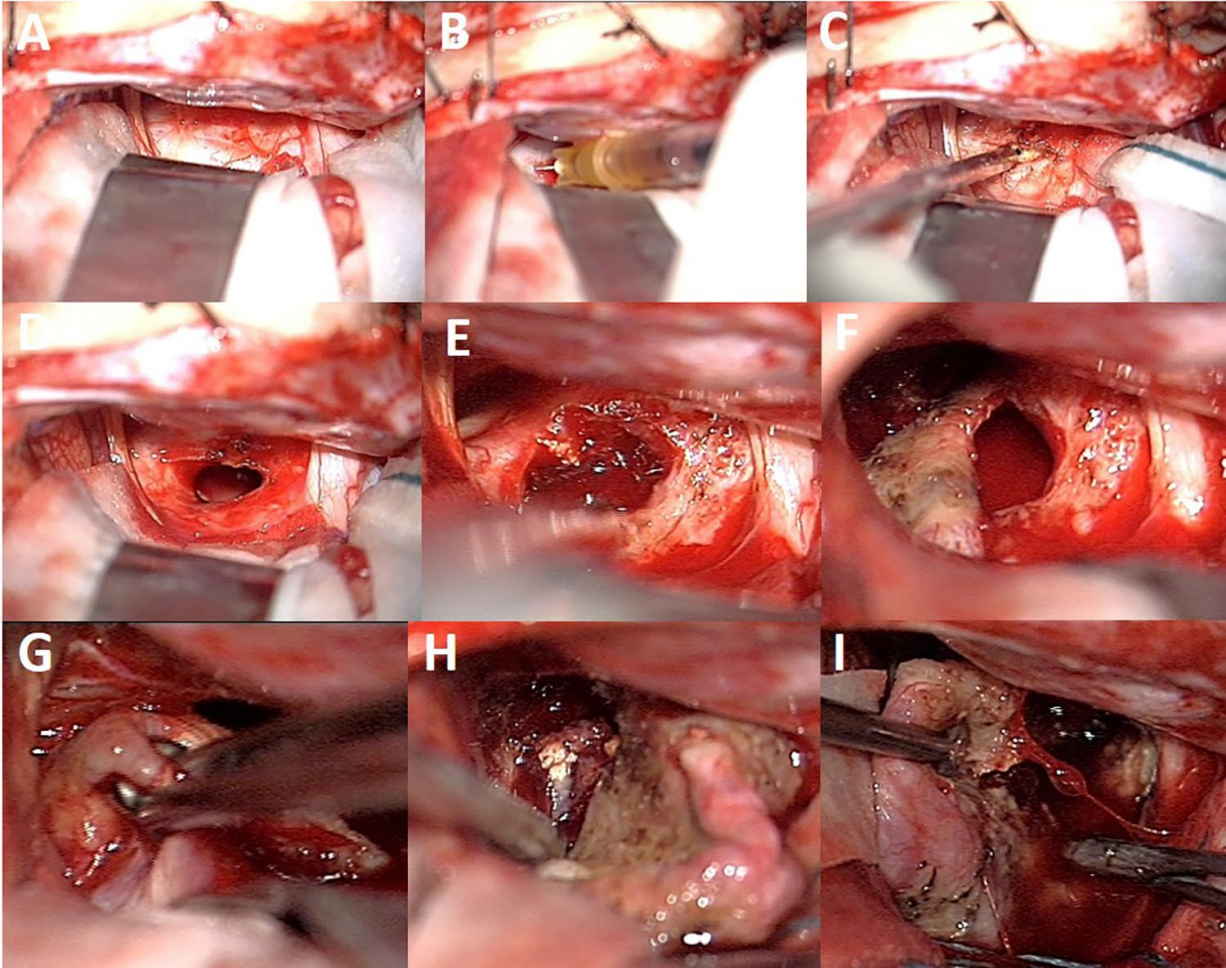
Intraoperative view of the same case in Fig. 2 (the child was operated when she was 8 years old, 3 years after the IA treatment, because of initial tumor-regrowth). In spite of the subtraction of the cystic fluid (**A**, **B**, **C**), the tumor capsule remains somewhat fibrotic, thicker, and more difficult do detach from the surrounding structures, and it does not deflate after the cyst fluid removal (**D**, **E**, **F**). This makes the tumor excision more complicated (**G**, **H**, **I**)

Apart from some minimal variants, the protocol for IA administration (given through an Ommaya reservoir and an intracystic catheter) is based on 3 million International Units (IU) of interferon-α-2a every other day for a total of 12 administrations per cycle (36 million IU), the cycle being repeated 1 month after the end of the previous one for 3 or more times [103]. The first multicenter study, provided by Cavalheiro et al. on 2010, recruited 60 pediatric patients from 2000 to 2009 and showed a tumor control in 47 patients (78.3%), with no mortality and very low morbidity rates [172]. More in details, 30% of children had minor side effects (like headache, fever, palpebral edema, fatigue, arthritis) that were easily controlled and disappeared after treatment. The follow-up ranged from 4 to 84 months: 13.3% of patients developed new endocrinological deficits, and the tumor control was obtained in 78.3% patients (13 children needed new surgery) with no differences in the disease course based on whether the patient had undergone previous treatments or not. The international multicenter cooperative study by Kilday et al., published in 2017 on 56 children (median age: 6.3 years), only 23% of whom received IA as a first treatment, showed the following results after 5.1-year median follow-up: (1) 75% of patients had a clinical or radiological progression during the follow-up even though 33% of them did not require any treatment for this progression, and (2) 41% of patients did not present adverse events [173]. The remaining children experienced influenza-like malaise (29%), headaches (18%), fatigue (13%), transient hyponatremia (2%), appetite loss (2%), and weight loss (2%). None of these symptoms was severe: (3) two cases of brain toxicity were registered (optic pathway edema and brain atrophy with hydrocephalus) due to the suspected spillover of IA in the subarachnoid spaces. Accordingly, the use of IA was encouraged to delay AC progression, thus postponing surgery or RT radiotherapy (which is crucial for children during the development age). Sometimes, IA is able to efface completely the cyst while, sometimes, just to shrink it (Fig. 2), in any case allowing to postpone surgery and to gain time for the child development. A possible limitation of IA is the thickening of the tumor wall after the treatment that can be often observed during surgery, which can make its removal more difficult (Fig. 3).

The main limitation of the use of intracystic IA is the current unavailability of the drug. Actually, due to the update of international regulation on the use of drugs, such a precious tool has been recently withdrawn from the market. Therefore, the interest could be addressed to the administration of subcutaneous pegylated IA (PIA). Yeung et al. proposed a protocol based on 1–3 μg/kg/week of PIA for 2 years [174]. Goldman and coworkers recently reported on 23 children and young adults with unresectable craniopharyngioma who were treated by the aforementioned protocol for 108 weeks [175]. The patients were divided in two groups: the first one included patients previously treated with surgery while the second one was composed by patients who had received radiation therapy. 28.8% of patients of the first group showed a partial response to the treatment but none of the second group presented a radiographically significant response. The progression free-survival was 17.7 and 19.5 months, respectively. As expected, the systemic toxicity of PIA was higher than intracystic IA, although only grade 2 and 3 hematologic toxicities occurred (no grade 4 or 5).

### Basic research

Under a biologically and molecular point of view, AC is a surprisingly rich tumor. The dysregulation of WNT/B-catenin pathway, necessary for the organ formation during the embryogenesis, for maintaining the stem cells in adulthood and controlling gene transcription and cell adhesion and migration, is typical of AC, being observed in 57–96% of cases [176]. Both cystic and solid components express various cytokines, chemokines, and inflammatory mediators which can be involved in cancer cells activities and play a promising role not only to explain the tumor development and progression but also to find possible therapeutic targets. For example, the preoperative high levels of α-defensins found in the fluid of AC could indicate a role of inflammation in stimulating the secretion of cyst fluid by the epithelial cells of the cyst wall, whilst the decrease of defensins in the postoperative period would account for the reduced fluid production and the cyst shrinkage [177]. Moreover, the treatment with IA has been found to reduce the defensins levels through an antitumoral effect on the squamous epithelial cells, an immuno-modulatory action on the recruitment of inflammatory cells, and an anti-angiogenic activity, further supporting the previous, inflammatory hypothesis [177]. Furthermore, AC was found also to present high immunoreactivity response to retinoic acid receptors (RARs), which have a role in cell maturation and differentiation and could be responsible, at least in part, of the recurrence of AC according to the expression of specific cathepsins (which are important for the cell turnover) [93, 105, 178]. More in details, according to Lubansu et al., recurrent AC show low levels of RAR-beta and high levels of RAR-gamma: AC with low RAR-beta express low levels of cathepsin D while AC with high RAR-gamma express high levels of cathepsin K [92]. Considering other factors and pathways, PD-1/PD-L1 expression, SHH signaling upregulation, BMP and MMP pathways, and BRAF mutations can represent potential goals for target treatment in AC [90, 179, 180], thus explaining the increasing number of trials on AC [181].

Actually, in the current era, which is more and more searching for target therapies, the results of the base research on AC are advocated to possibly develop new therapeutic agents (both intracystic and systemic) which could interfere with AC molecular pathways. As mentioned, the inflammatory mediators seem to be promising in AC since they are involved in cancer cell activities (tumor development and progression) and could be targeted for possible therapies. IL-6 pathway is an example of a pathway showing target drugs that have been successfully used in AC [182, 183]. Tocilizumab (an anti-IL6 monoclonal antibody) has been systemically administered to two patients with recurrent AC after cyst aspiration and RT [95, 184, 185]. The preliminary results showed that one patient had a tumor volume reduction and stability without side effects after a 7-month cycle and the other one had to interrupt the first cycle after 8 months because of disease progression. It is worth noting that, in this second patient, the tumor control was obtained afterwards by a combination of tocilizumab and bevacizumab (an anti-angiogenetic, anti-VEGF monoclonal antibody). Neutropenia is the main side effect of this treatment.

Further, potential targets could come from the analysis of the clonal mutations that drive the oncogenesis of craniopharyngioma [186, 187]. AC is characterized by alterations in the Wnt/b-catenin pathway, mainly involving the central regulatory gene CTNNB1, while papillary craniopharyngioma is mostly driven by the V600E mutation in the BRAF gene, which activates the mitogen-activated protein kinase (MAPK) signaling pathway [186–189]. These molecular changes could disclose potential targets for new therapeutic approaches to improve long term tumor control volume and to decrease the associated morbidity [190, 191]. Calvanese et al. reported two cases of adult patients with papillary craniopharyngioma who underwent anti-BRAF/MEK combined therapy as adjuvant (case 1) or neoadjuvant (case 2) treatment [192]. Case 1 underwent a SR through EETA (the tumor harbored the BRAF V600E mutation) and a subsequent adjuvant target therapy with Dobrafenib and Trametinib with a 40% reduction in tumor volume after 2 months and a 90% reduction after 5 months. Case 2 underwent a transventricular endoscopic biopsy and, afterwards, started the same combined therapy with a 80% reduction in tumor volume after 2 months and a 90% reduction after 4 months. Hopefully, similar treatments will be available also for AC.

## Personal experience: results of the paradigm shift

All children with AC consecutively treated at our institution between January 1985 and July 2021 have been considered for the present report to briefly summarize the personal model shift in the management of such a challenging tumor.

### Patients and methods

Only children with comprehensive preoperative and postoperative work-up and with complete, 2-year minimum follow-up were considered for the present study. Ninety-three children were therefore enrolled among 114 patients composing the whole AC series: 52 patients belonged to the “old era” (group 1: 1985–2003), where the goal of treatment was to achieve GTR, while 41 patients to the “new era” (group 2: 2004–2021), where a more conservative, multimodal approach was used including SR/PR, RT and intra-cystic IA administration. The statistical analysis was done by using the chi-squared test and by setting the significance with a *p* value < 0.05.

Group 1 consists of 33 boys and 19 girls, with a 9-year mean age at surgery (ranging from 20 months to 16 years). The main clinical findings were increased intracranial pressure signs/symptoms (62% of cases), growth retardation or low stature (44%), deterioration of visual acuity (25%), hemianopia or other visual filed restrictions (17%), asthenia and hypotension (21%), diabetes insipidus (19%), hypo/anorexia and weight loss (17%), and hyperphagia and weight (6%). Preoperative panhypopituitarism was detected in 10 cases (19%) and selected hormone deficits in 14 cases (27%). Neuroradiological work-up included CT scan and/or MRI: AC was mainly cystic in 24 cases (46%), mainly solid in 8 (16%), and solid-cystic in 20 (38%); calcifications were detected in 32 cases (62%). The purely intrasellar location was evident only in 3 cases (6%), while the remaining children harbored a sellar/suprasellar prechiasmatic AC (24 cases, 46%) or a sellar/suprasellar retrochiasmatic AC (14 cases, 27%) or a giant AC (11 cases, 21%). Obstructive hydrocephalus at the time of diagnosis was present in 17 cases (33%).

Group 2 is composed by 24 boys and 17 females, with a mean age at surgery of 8.5 years (range: 12 months–17 years). The main clinical manifestations were related to raised intracranial pressure in 61% of cases, growth retardation or low stature in 17%, asthenia and/or hypotension in 12%, diabetes insipidus, delayed sexual development, weight loss and weight gain in 7% each, visual disturbances (including reduction of visual acuity, cranial nerves deficits and strabismus, visual field restriction and nystagmus) in 85%, hemiparesis and gait ataxia in 9.5% each, behavioral disturbances in 5%, and dysregulation of the sleep rhythm in 2.5%. Preoperative laboratory investigation revealed a panhypopituitarism in 2 cases (5%) and partial hormone deficits in 17 patients (41%). All patients received both CT scan and MRI. A mainly cystic AC was evident in 9 cases (22%), a mainly solid one in 10 cases (24%) and a solid-cystic one in 22 (54%); calcifications were detected in 15 cases (37%). Considering the tumor location, AC was purely intrasellar in 4 patients (10%), sellar/suprasellar with prechiasmatic growth in 24 cases (58%), sellar/suprasellar with retrochiasmatic growth in 10 cases (24%), and giant in 3 cases (8%). Hydrocephalus was present in 16 cases (39%).

### Results

In group 1, surgery was realized through a single approach in 47 patients (90%) and a combined approach in the remaining 5 (10%). More in details, 41 patients underwent a TMA alone, 6 children were operated via transsphenoidal sublabial route, 4 children underwent a transsphenoidal approach followed by craniotomy and one child a craniotomy followed by a transsphenoidal approach. GTR was achieved in 40 cases (77%), SR in 17%, and PR in 6%. The associated hydrocephalus required a separate surgical operation in 7 out of 17 cases. Two surgery-related deaths (3.8%) occurred in this group, both following a transsphenoidal sublabial procedure: the first one as a consequence of a massive intra-operative bleeding in an attempt to remove a calcification on the lateral wall of the sella, the second one as a consequence of a gram-negative CSF infection complicated by cerebral venous sinus thrombosis. The most common mechanical complication was represented by postoperative subdural collection in 8 cases, requiring a drainage in 3 cases. Visual deficits improved in 40% of cases but worsened in 36%, and neurological deficits improved in 64% of cases (Table 3).

**Table 3:** Comparison between the two groups about visual e neurological outcome

***Type of deficit***	***Postoperative outcome***	***Group 1***	***Group 2***	***p value***
Visual	Improved	40%	54%	Not significant
	Stable	24%	34%	Not significant
	Worsened	36%	12%	0.007
Neurological	Improved	64%	85%	0.01
	Stable	36%	15%	0.02

Nine children in group 1 presented a tumor recurrence/regrowth at variable time interval from the surgical treatment (17%). In 3 of them (6% of the whole group), a true recurrence after GTR occurred; in the remaining cases, it was about a re-growth of residual tumor left behind at operation. All the recurrences were treated surgically: GTR was achieved in five cases and SR/PR 4 cases. One postoperative death was recorded (surgical mortality for recurrence: 10%).

RT was administered postoperatively to 12 children of this group (23%); only one of them developed recurrence after the treatment. The tumor control was obtained in 92% of cases. One boy experienced the novel development of a malignant thalamic glioma 8 years after the completion of RT and required new surgery and chemotherapy.

The mean follow-up of this group was 17.5 years. After this period, 46 patients were alive (88%). Other than the 2 surgical mortalities, the causes of death were late post-RT malignancy (1 case), tumor progression (2 cases), and late systemic complications (1 case). The residual visual deficit did not prevent all but 3 patients from attending standard school. Two patients presented permanent hemiparesis, and 2 needed anti-epileptic drugs for postoperative epilepsy. Forty-three percent of patients required treatment for diabetes insipidus, 80% developed panhypopituitarism requiring hormone replacement, and 23% developed hypothalamic obesity (Table 4).

**Table 4:** Summary of the comparison between the two groups about tumor control and hypothalamic outcome

	***Group 1***	***Group 2***	***p value***
Rate of recurrence	6%	15%	Not significant
Surgical-related mortality	3.8%	0%	Not significant
Overall survival	88%	93%	Not significant
Panhypopituitarism	80%	24%	< 0.00001
Hypothalamic obesity	23%	7%	0.04

**Fig. 4:**
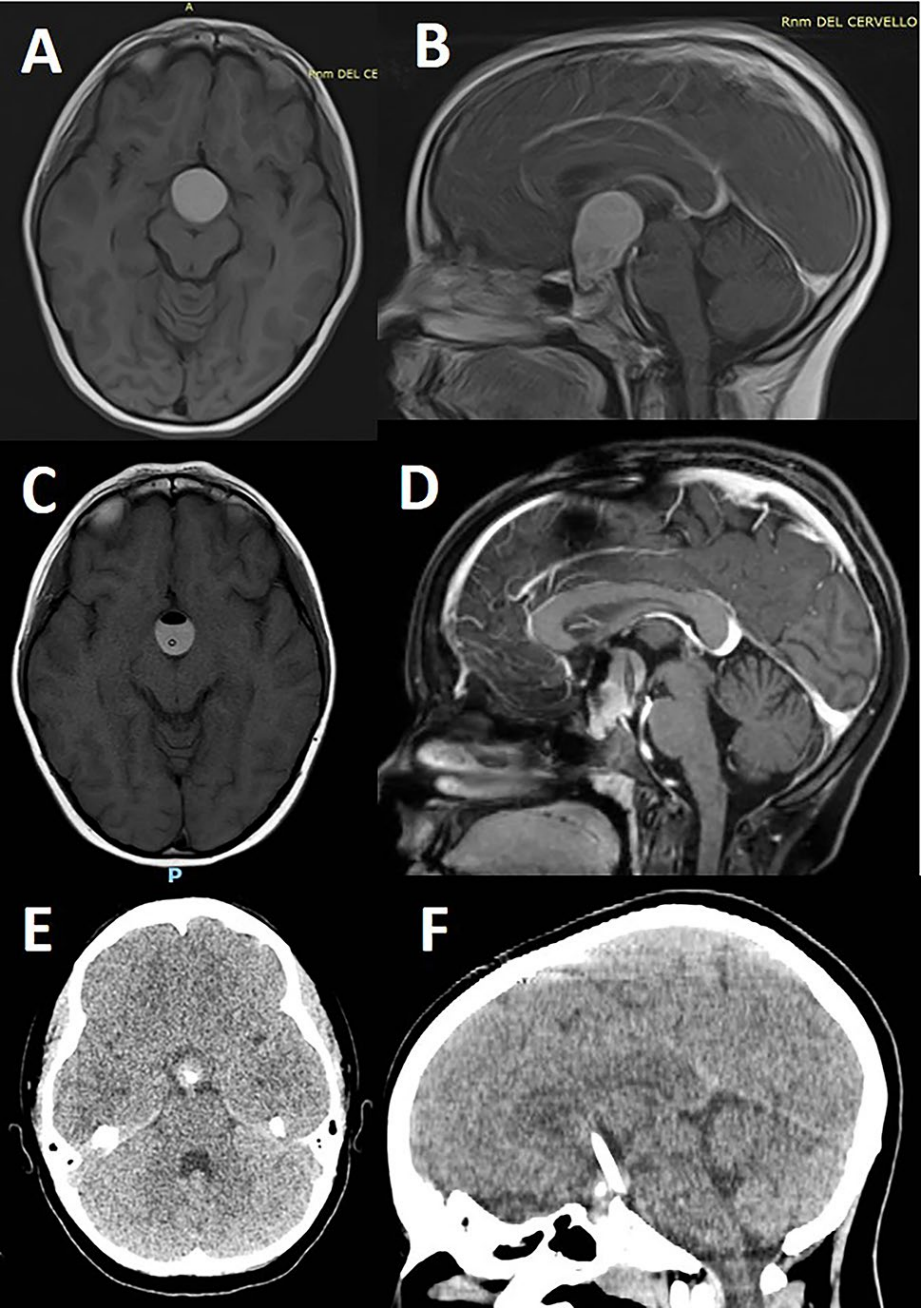
T1 MRI before (**A**) and after gadolinium administration (**B**) showing the presence of AC in a 7-year-old girl; **C**, **D** the same MRI sequences after navigation-guided placement of intracystic catheter and aspiration of motor-oil fluid, which allowed a significant reduction of the cyst with decompression of the surrounding structures; **E**, **F** CT scan performed at the last follow-up (4 years later and after 3 cycles of IA) showing the effacement of the cystic component with evidence of a small, calcified intrasellar, tumor solid portion

In group 2, 17 patients (41%) underwent surgical treatment by TMA and 24 (59%) by EETA. GTR was obtained in 17 patients (41%), SR in 15%, and partial in 12%. 32% of patients received the implantation of an intracystic catheter plus subcutaneous reservoir for cyst aspiration (in 3 cases) and to deliver IA (in the remaining 10 cases) (Fig. 4). Hydrocephalus had to be surgically addressed in 4 out of 16 affected children. No surgery-related deaths were recorded in this group. Nine patients developed a subdural hygroma; one of them required surgical drainage and another required a permanent subdural peritoneal shunt system. In the early period, visual deficits were improved in 54% of patients and worsened in 12%. Neurological deficits were improved in 85% of them (Table 3).

In group 2, 6 children showed a tumor recurrence after GTR (15%), while 20 children had a tumor re-growth (48%). Recurrences were treated surgically in a single procedure in 14 cases, in 2 steps in 6 cases, in 3 or more steps in 6 cases. No postoperative deaths occurred.

RT was administered postoperatively to 8 patients of this group (19.5%). Three of them developed recurrence after the treatment: therefore, the disease was controlled in 63% of them. Also, in this group, one patient developed a malignant thalamic glioma 10 years after the completion of RT and required radiation therapy and chemotherapy.

All but 3 patients (93%) are still alive after a 8.5-year mean follow-up. One patient died because of tumor progression, one patient because of complication of obesity surgery and one patient because of late RT-induced malignancy. Neurological and visual deficit improvement was demonstrated in up to 80% of patients compared with the diagnosis. Twenty-four percent of patients developed panhypopituitarism requiring hormone replacement, and 7% developed hypothalamic obesity (Table 4).

### Summary

In synthesis, the personal experience confirms the advantages of the multimodal treatment in reducing the late sequelae of AC management. Indeed, the comparison between the two groups shows a statistically significant reduction of visual worsening and a relevant increase of neurological improvement during the modern era compared with the past one (Table 3). Moreover, both the occurrence of panhypopituitarism and obesity were significantly reduced in the postoperative period by the multimodal treatment (Table 4). These results were obtained without relevant differences between the two groups in terms of tumor recurrence and overall survival.

## Conclusions

Both the historical excursus with the review of the literature and the personal experience suggest that radical surgery allows for a good AC control with a lower rate of recurrence, but it is burdened by relatively high risk of mortality and significant, permanent morbidity as far as visual, neurological and endocrine outcomes are concerned. For this reason, a more conservative policy has been recently adopted that, despite the greater number of recurrences and surgeries, seems to be safer both in terms of complications and mortality: the higher recurrence risk is balanced by better clinical conditions, preservation of endocrine functions, and minor development of hypothalamic obesity. In current times, where the QoL has gained more and more importance so that it is often considered by patients more relevant than the survival itself, the efforts made to enhance the multimodal management of AC seem to be the best choice to preserve QoL. Actually, the improvement in EETA and neuroendoscopy, in the proton RT and in the management of the cyst offers the pediatric neurosurgeon a great possibility to treat AC both with good oncological and functional outcomes: the key point is to use these therapeutic options together and not singularly. AC is still a challenging tumor and an ideal treatment is not yet available but, hopefully, the results coming from the basic research could enrich the multimodal treatment up to win such a challenge.
